# Prevalence and Clinical Implications of Somatic and Germline EGFR Mutations in Patients with Non-Small-Cell Lung Cancer

**DOI:** 10.3390/cancers18152417

**Published:** 2026-07-27

**Authors:** Jingyao Zhang, Linjun Zha, Ruqiang Liang, Tianhong Li

**Affiliations:** 1 Department of Internal Medicine, Danbury Hospital, Zucker School of Medicine at Hofstra/Northwell, Danbury, CT 06810, USA; jingyao.zhang@nuvancehealth.org; 2 Division of Hematology/Oncology, Department of Internal Medicine, University of California Davis School of Medicine, University of California Davis Comprehensive Cancer Center, Sacramento, CA 95817, USA; lzha@health.ucdavis.edu (L.Z.); rqliang@health.ucdavis.edu (R.L.); 3 Medical Service, Hematology/Oncology, Veterans Affairs Northern California Health Care System, Mather, CA 95655, USA

**Keywords:** epidermal growth factor receptor, somatic mutations, germline mutations, non-small cell lung cancer, lung adenocarcinoma, targeted therapy, precision medicine, risk stratification, screening, prevention

## Abstract

Epidermal growth factor receptor (EGFR)-targeted therapy represents one of the earliest and most established examples of precision oncology in non-small-cell lung cancer (NSCLC), with more than 10 approved agents, including tyrosine kinase inhibitors, bispecific antibodies and antibody–drug conjugates. Over the past two decades, the diagnostic and therapeutic landscape of EGFR-mutant NSCLC has evolved from empiric treatment to mutation subtype-guided strategies, from advanced disease to earlier-stage interventions, and from monotherapy to rational combination regimens. Somatic EGFR mutations remain key predictive biomarkers guiding treatment selection, therapeutic intensification, resistance mechanism-directed treatment, and disease monitoring through plasma circulating tumor DNA burden. In parallel, germline EGFR alterations are increasingly recognized as contributors to inherited lung cancer susceptibility, particularly among never-smokers and familial clusters. Germline EGFR T790M is the best-characterized pathogenic variant, creating a permissive background for multifocal lung nodules and lung adenocarcinoma development, often following acquisition of a second somatic EGFR driver mutation. Recent familial, regional, and paired tumor–normal sequencing studies have expanded the evidence beyond isolated case reports and support an emerging hereditary lung cancer predisposition phenotype. Clinically, germline EGFR should be suspected when EGFR T790M is detected prior to TKI exposure, particularly at variant allele fractions near 50%, or in patients with multifocal ground-glass nodules, multiple primary lung adenocarcinomas, early-onset disease, never/light smoking history, or family history of lung cancer. Confirmation requires germline testing and genetic counseling. This review highlights the current knowledge, recent advances, and future directions in somatic and germline EGFR-mutant NSCLC, emphasizing translational relevance for clinicians and researchers.

## Simple Summary

Epidermal Growth Factor Receptor (EGFR) is frequently overexpressed or mutated in epithelial-derived tumors. In non-small-cell lung cancer (NSCLC), activating EGFR mutations in the cytoplasmic tyrosine kinase domain are important therapeutic targets. EGFR-targeted therapy was among the earliest successful precision-medicine approaches in lung cancer and remains a well-established example of precision oncology, particularly for women, never-smokers, and patients with adenocarcinoma. Between 2014 and 2024, treatment strategies evolved from unselected approaches to EGFR mutation subtype-guided therapies, from advanced unresectable disease to early-stage resected disease, and from monotherapy to combination regimens. The therapeutic armamentarium has expanded beyond small-molecule tyrosine kinase inhibitors to include bispecific antibodies and antibody–drug conjugates. While somatic EGFR mutations guide tumor-directed treatment and resistance assessment, germline EGFR mutations are increasingly recognized for their potential contribution to inherited lung cancer susceptibility and family risk assessment. However, their population prevalence, penetrance, and optimal surveillance remain uncertain. This review summarizes recent advances and future directions in the management of EGFR-mutant NSCLC.

## 1. Introduction

Epidermal growth factor receptor (EGFR) is a transmembrane receptor tyrosine kinase expressed on epithelial cells and frequently dysregulated in epithelial malignancies, including non-small-cell lung cancer (NSCLC), head and neck squamous-cell carcinoma, glioblastoma, and colorectal cancer [1,2,3]. EGFR signaling regulates cellular proliferation, differentiation, migration, survival, angiogenesis, and metastasis. Aberrant EGFR activation can occur through receptor overexpression, autocrine ligand stimulation, and constitutively activating receptor mutations; EGFR signaling can also be transactivated through crosstalk with other receptor networks, including G protein-coupled receptor signaling pathways [4].

Gain-of-function somatic mutations in the EGFR tyrosine kinase domain define the first molecularly selected subset of NSCLC patients to derive substantial benefit from targeted therapy. These somatic EGFR alterations are particularly enriched in female and lung adenocarcinoma and demonstrate marked variability according to geography, ancestry, smoking history, and disease setting [5,6,7,8,9,10]. In advanced NSCLC, classic activating EGFR mutations, most notably exon 19 deletions and exon 21 L858R substitutions, predict substantial clinical benefit from EGFR tyrosine kinase inhibitors (TKIs), including erlotinib, gefitinib, afatinib, dacomitinib, and osimertinib, compared with platinum-based chemotherapy, fundamentally reshaping first-line treatment paradigms [11]. With the widespread adoption of next-generation sequencing (NGS), the genomic landscape of EGFR-mutant NSCLC has become increasingly well characterized. EGFR exon 19 deletions (40–50% of cases) and exon 21 L858R substitutions (30–40%) constitute the two major types of somatic EGFR mutations, often referred to as common (classic) mutations. The remaining 10–15% are classified as uncommon EGFR mutations and include exon 20 insertions (approximately 5%), as well as atypical alterations involving exons 18–21, such as exon 18 G719X, exon 20 S768I, and exon 21 L861Q. Approximately 14% of NSCLC harboring uncommon EGFR mutations also carry additional EGFR alterations, either common or uncommon; these are collectively termed compound EGFR mutations. These distinct EGFR genotypes have important clinical implications for treatment selection, therapeutic intensification, resistance mechanism-directed strategies, and disease monitoring using plasma circulating tumor DNA (ctDNA), which are discussed in subsequent sections.

In parallel, germline EGFR alterations are increasingly recognized as contributors to inherited lung cancer susceptibility, particularly among never-smokers and familial cancer clusters. In contrast to somatic EGFR mutations, which function as acquired oncogenic drivers, germline EGFR variants are rare inherited alterations that predispose individuals to familial lung cancer syndromes. Among germline variants, EGFR T790M is the best-characterized pathogenic alteration in lung cancer tumorigenesis. Table 1 summarizes the key clinical and biological distinctions between somatic and germline EGFR mutations.

These two categories have complementary but distinct clinical implications. Somatic EGFR alterations are tumor-specific biomarkers that guide systemic treatment selection, resistance assessment, and disease monitoring. In contrast, a confirmed germline EGFR pathogenic variant primarily informs hereditary-risk assessment, genetic counseling, cascade testing, and individualized surveillance discussions. Germline status generally should not determine systemic therapy independently of the tumor’s somatic driver, disease stage, and clinical context.

Although numerous reviews have summarized recent therapeutic advances in somatic EGFR-mutant NSCLC [12,13,14,15,16,17], few have comprehensively addressed both somatic and germline EGFR alterations. This review integrates current evidence on their biological significance, therapeutic implications, and translational relevance, with particular emphasis on the emerging role of germline EGFR mutations in lung cancer susceptibility, screening, prevention, and clinical management.

## 2. Somatic EGFR Mutations

### 2.1. Global Prevalence of Somatic EGFR Mutations

**Figure 1:**
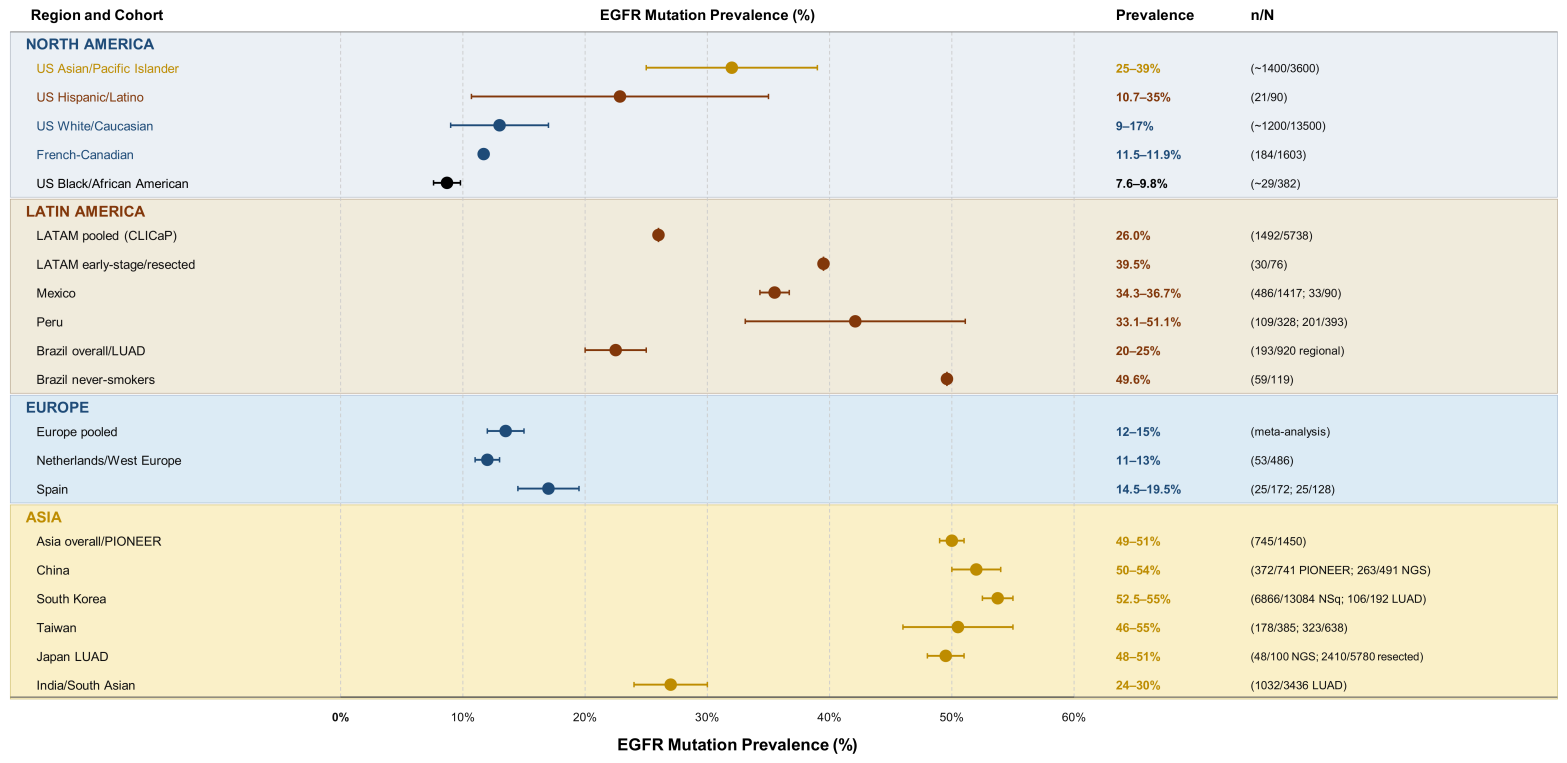
Regional and Ethnic Differences in EGFR Mutation Prevalence Among Tested Patients with NSCLC. EGFR mutation prevalence varies substantially according to geographic region, ethnicity, histologic subtype, disease stage, and molecular testing platform. The values shown represent selected published estimates from tested NSCLC or lung adenocarcinoma cohorts by NGS. These estimates are derived from heterogeneous cohorts that differ in histologic composition, disease stage, smoking-status enrichment, patient selection criteria, and testing methodology; therefore, direct cross-regional comparisons should be interpreted with caution and do not represent standardized population-based incidence rates. Detailed cohort characteristics, sample sizes, assay methods, and selection criteria are provided in Supplementary Table S1.

The prevalence of somatic EGFR mutations in NSCLC varies substantially by ethnicity and geographic region and the clinical population studied. Across broad, tested NSCLC cohorts worldwide, the pooled prevalence of EGFR mutations has been estimated at 32.3%. A subsequent global meta-analysis reported prevalences of 49.1% among Asian NSCLC cohorts and 12.8% among European NSCLC cohorts. Consistent with these findings, the PIONEER study identified EGFR mutations in 51.4% of Asian patients with advanced lung adenocarcinoma [5,6,7]. Regional estimates vary substantially, including approximately 46–55% in recent Taiwanese NSCLC or lung adenocarcinoma cohorts, 23–34% in broad Indian NSCLC or lung adenocarcinoma cohorts, 34–37% in Mexican NSCLC datasets, 20–25% in overall Brazilian NSCLC or lung adenocarcinoma cohorts but nearly 50% among Brazilian never-smoker lung adenocarcinoma cohorts, 10.7–35% among U.S. Hispanic/Latino NSCLC cohorts, and approximately 12–15% across broad European NSCLC datasets [18,19,20,21,22,23,24,25,26,27,28,29,30,31,32,33,34,35,36,37,38,39,40,41,42,43,44,45,46,47,48,49,50,51,52]. Genetic ancestry also contributes to this variation; in admixed Latin American lung cancers, Indigenous American ancestry was associated with higher EGFR mutation frequency independent of smoking and sex [33]. However, the underlying denominators vary across studies and include broad NSCLC cohorts, adenocarcinoma-only populations, early-stage or advanced disease, never-smoker-enriched cohorts, and selected molecular-testing populations. These values therefore represent the prevalence or frequency of somatic EGFR mutations among tested clinical cohorts rather than the population incidence of EGFR-mutant NSCLC and should be interpreted in the context of histology, smoking status, disease stage, ancestry, patient selection, and testing platform. Detailed cohort characteristics are summarized in Supplementary Table S1. Although NGS is not widely available for patients with NSCLC in Vietnam, Nguyen and colleagues reported EGFR mutations in 26 of 45 (57.8%) NSCLC tumors diagnosed in Hue, Vietnam, and in 21 of 44 (47.7%) Vietnamese patients with NSCLC treated at Stanford University [53]. These findings highlight persistent barriers to comprehensive molecular testing, including limited affordability and access to biomarker-directed therapies. Figure 1 summarizes selected published regional and ancestry-associated estimates of somatic EGFR mutation prevalence [8,9,10,54]. The figure is intended to illustrate the reported range and should not be interpreted as a direct cross-cohort or cross-region comparison.

### 2.2. Clinical Implications of EGFR-Targeted Therapies Based on Somatic EGFR Mutations

The College of American Pathologists (CAP), the International Association for the Study of Lung Cancer (IASLC), and the Association for Molecular Pathology (AMP) first issued molecular testing guidelines in 2013 for selecting lung cancer patients for EGFR and ALK TKIs in all advanced LUAD patients regardless of clinical characteristics, such as age, race, and smoking status [55]. NGS enables comprehensive characterization of the heterogeneous spectrum of EGFR genomic alterations in NSCLC, including common sensitizing (“classic”) mutations, uncommon or atypical EGFR mutations, EGFR exon 20 insertion (Ex20ins) mutations, and P-loop and αC-helix compressing (PACC) mutations, each with distinct biological behavior and clinical benefit to EGFR-targeted therapies (Table 2) [56,57]. Currently, testing for classic EGFR somatic mutations is recommended for all stages of non-squamous NSCLC, regardless of clinical characteristics [55,56]. Table 2 summarizes available treatment options according to the EGFR mutation subtype and treatment setting according the FDA approval.

Emerging data support distinct clinicopathologic features and therapeutic responses associated with uncommon EGFR mutations. In patients with early-stage EGFR-mutant NSCLC, uncommon EGFR mutations were associated with shorter disease-free survival (DFS), central nervous system DFS, and overall survival (OS) than EGFR exon 19 deletions or L858R mutations, with EGFR exon 20 insertions (Ex20ins) conferring particularly poor outcomes [58]. Real-world analyses across all stages of EGFR-mutant NSCLC have demonstrated distinct clinicogenomic characteristics and inferior OS among patients with uncommon compared with common EGFR mutations [59]. Therapeutic advances are also emerging for specific subgroups of uncommon EGFR mutations as discussed below.

#### 2.2.1. EGFR TKI Monotherapy

**Figure 2:**
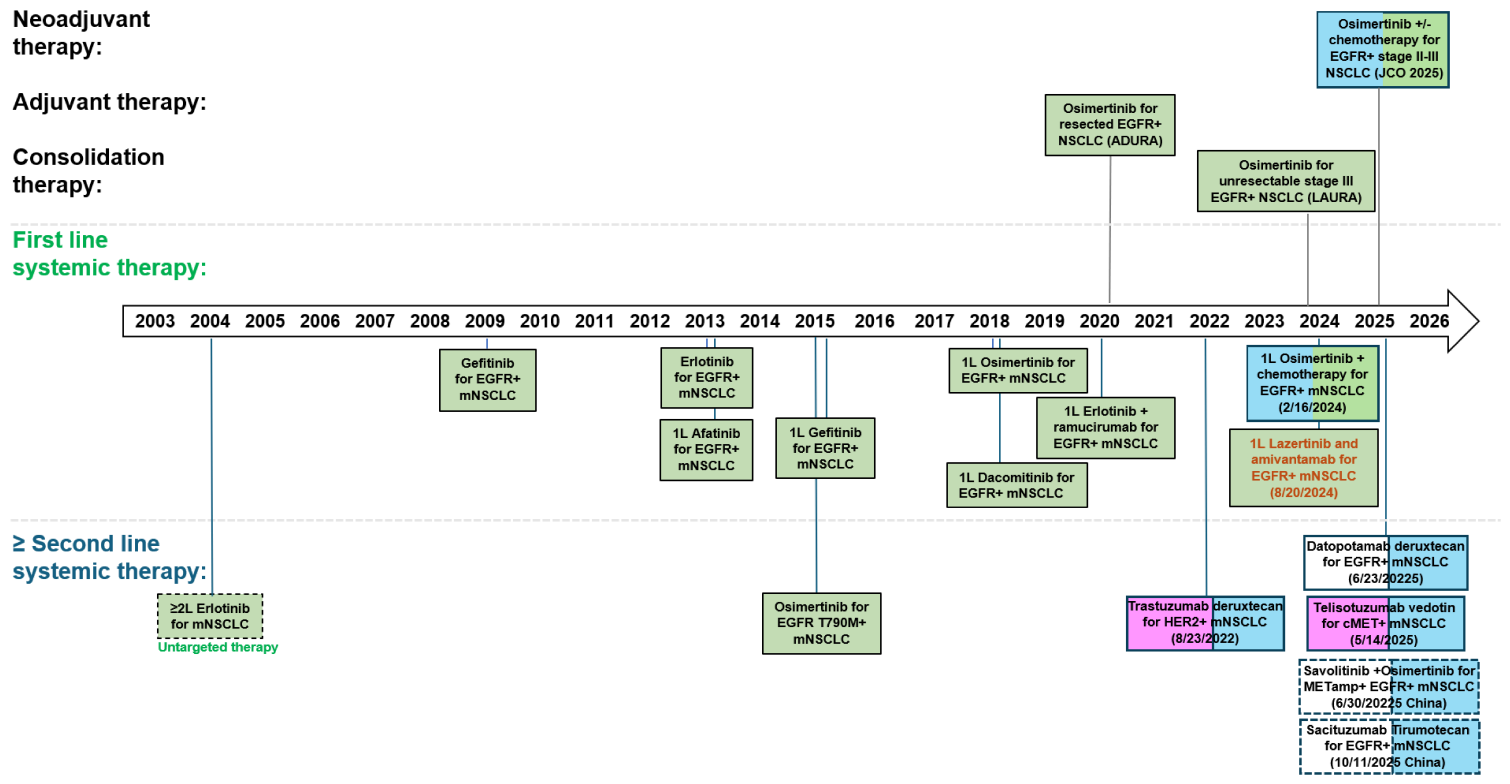
The evolving treatment landscape for classic EGFR-mutated NSCLC. Over the past few decades, many advances have contributed to the improved OS for patients with nonresectable, LA/mNSCLC, which includes first-line platinum-based combination chemotherapy, second-line single-agent chemotherapy or unselected molecularly targeted therapy, histology-directed chemotherapy, and tumor genotyping for molecular biomarkers, and first- and second-generation molecularly targeted therapies in the United States. Notably, patients with metastatic lung adenocarcinoma have benefited most from these therapeutic advances. Green, EGFR-directed targeted therapies; pink, other targeted therapies; blue, chemotherapy-containing regimens. Dashed box, not biomarker-guided therapy or not US FDA approved drugs.

The development of EGFR TKIs has transformed the treatment landscape of EGFR-mutant NSCLC (Figure 2). Erlotinib, a first-generation EGFR TKI, was the first molecularly targeted therapy to receive FDA approval in 2004 as second-line treatment for unselected NSCLC patients [60]. Subsequently, gefitinib, afatinib, dacomitinib, icotinib, and osimertinib expanded the therapeutic armamentarium for patients with the two classic common EGFR mutations. Afatinib, a second-generation irreversible ErbB family blocker, received FDA approval in 2013 for first-line EGFR-mutant NSCLC [61]. In January 2018, its indication was broadened to include three uncommon EGFR mutations (G719X, L861Q, and S768I), making afatinib the EGFR TKI with the broadest FDA-approved first-line indication among EGFR-mutant NSCLC subtypes [62].

The third-generation EGFR inhibitor osimertinib was initially introduced as a second-line therapy for patients treated with earlier generation EGFR TKIs who developed the T790M resistance mutation [63]. Subsequently, osimertinib has emerged as the preferred frontline therapy for patients with classic EGFR mutations, particularly exon 19 deletions and exon 21 L858R substitutions. In the landmark FLAURA trial, first-line osimertinib monotherapy demonstrated superior efficacy compared with first-generation EGFR TKIs (gefitinib or erlotinib), achieving a median progression-free survival (mPFS) of 18.9 months versus 10.2 months and a mOS of 38.6 months versus 31.8 months while maintaining a favorable toxicity profile and robust central nervous system (CNS) activity [64]. Overall, these classic EGFR mutations are highly sensitive to EGFR TKIs, achieving objective response rates (ORRs) of 60–80% and mPFS ranging from 9 to 19 months [65,66]. Osimertinib has been endorsed as a preferred first-line monotherapy in clinical practice guidelines [67]. Osimertinib has also been incorporated into curative-intent treatment strategies in classic EGFR-mutant NSCLC. As adjuvant therapy, osimertinib has demonstrated substantial improvements in DFS and OS in patients with resected stage IB–IIIA disease [68,69]. In the neoadjuvant setting, osimertinib, with or without chemotherapy, has achieved significantly higher major pathological response rates compared with chemotherapy alone, but the pathological complete remission rates remain low in patients with resectable stage II–IIIB N2 EGFR mutation-positive NSCLC [70]. In addition, consolidation therapy with osimertinib has significantly prolonged PFS compared with placebo in patients with unresectable stage III disease following definitive chemoradiotherapy [71].

For uncommon EGFR mutations, afatinib received FDA approval for three additional EGFR mutations (L861Q, G719X and S768I) in January 2018 [62]. Recent data have shown that osimertinib has an ORR of 55%, a mPFS of 9.4 months, and a median duration of response (DoR) of 22.7 months in rare, uncommon EGFR mutations other than exon 20 insertions (UNICORN) [72,73]. In patients with advanced NSCLC harboring atypical EGFR mutations (excluding exon 20 insertions), amivantamab plus lazertinib demonstrated clinically meaningful activity in Cohort C of the CHRYSALIS-2 study, a non-randomized, single-arm, phase I/Ib trial with a relatively small sample size. In the updated analysis of treatment-naïve patients presented at ASCO 2026, median overall survival (mOS) was 41.0 months, with 55% of patients alive at 3 years and no new safety signals observed during extended follow-up [74,75]. However, these findings should be considered hypothesis-generating at the absence of a randomized comparator, limited sample size, and still-maturing survival data.

However, EGFR exon 20 insertion mutations remain intrinsically resistant to conventional EGFR TKIs, including osimertinib, due to steric alterations in the kinase domain that impair drug binding. To address this unmet need, several targeted therapies have been developed. On 21 May 2021, the FDA granted accelerated approval to amivantamab-vmjw, a bispecific EGFR-MET antibody, for patients with locally advanced or metastatic NSCLC harboring EGFR exon 20 insertion mutations following progression on platinum-based chemotherapy [76]. Mobocertinib, an oral EGFR TKI specifically developed for exon 20 insertion mutations, also received accelerated approval in September 2021 for patients with disease progression after platinum chemotherapy; however, following failure to demonstrate a significant mPFS benefit in a phase III trial, it was voluntarily withdrawn from the U.S. market in October 2023 [77]. More recently, treatment options for EGFR exon 20 insertion NSCLC have expanded into the frontline setting. On 1 March 2024, the FDA approved amivantamab in combination with carboplatin and pemetrexed as first-line treatment for patients with locally advanced or metastatic NSCLC harboring EGFR exon 20 insertion mutations, representing a major advance for this historically difficult-to-treat population [78]. Subsequently, on 2 July 2025, the FDA granted accelerated approval to sunvozertinib, a next-generation oral EGFR TKI, for patients with locally advanced or metastatic NSCLC harboring EGFR exon 20 insertion mutations whose disease had progressed on or after platinum-based chemotherapy [79]. In this phase I/II WU-KONG1B study (NCT03974022), among 85 previously treated patients with metastatic NSCLC harboring EGFR exon 20 insertion mutations, sunvozertinib achieved an objective response rate (ORR) of 46% (95% CI, 35–57) with a median duration of response (DoR) of 11.1 months (95% CI, 8.2–not evaluable). The safety profile was generally manageable and consistent with EGFR inhibition, with the most common treatment-related adverse events including gastrointestinal toxicities (diarrhea, constipation, nausea, and vomiting), rash, dry skin, stomatitis, ocular toxicities, fatigue, and pneumonitis [79]. These findings supported its activity in the post-platinum setting, although interpretation remains limited by the single-arm design and relatively small sample size. In the randomized phase III WU-KONG28 trial, first-line sunvozertinib significantly improved progression-free survival and objective response rate compared with platinum-pemetrexed chemotherapy [80]. However, overall survival data remain immature, long-term safety follow-up is limited, and no direct head-to-head comparisons have been conducted against amivantamab-based regimens or other exon 20 insertion-directed strategies. Accordingly, cross-trial comparisons should be avoided, and the optimal positioning of sunvozertinib among available treatment options remains to be defined.

EGFR mutations are uncommon in squamous-cell lung carcinoma, with a reported prevalence of approximately 2–10%, although the true incidence in pure squamous tumors is likely lower (approximately 3–5%) and enriched among never-smokers, Asian patients, and tumors with mixed histologic features [81,82]. Thus, comprehensive NGS is still occasionally justified in squamous histology, especially in never-smokers or small biopsies, which aligns well with NCCN/IASLC practice [56,67,82]. Although afatinib remains the only FDA-approved EGFR TKI in unselected squamous NSCLC, a small subset of squamous tumors harbor activating classic EGFR mutations and may derive clinical benefit from EGFR-targeted therapy, albeit with generally lower response rates and shorter progression-free survival compared with EGFR-mutant adenocarcinoma [81,82,83]. In parallel, the anti-EGFR monoclonal antibody necitumumab, in combination with platinum-based chemotherapy, was approved in 2015 for patients with metastatic squamous NSCLC [84]. However, necitumumab-based chemotherapy has been infrequently adopted in routine clinical practice because of its modest clinical benefit, added toxicity, and the rapidly evolving therapeutic landscape of chemoimmunotherapy in squamous NSCLC [85].

#### 2.2.2. Combination Therapies for First-Line EGFR-Mutant NSCLC

Despite the survival benefit established with osimertinib monotherapy, considerable efforts have focused on combination strategies aimed at delaying resistance and further improving long-term outcomes in EGFR-mutant NSCLC. The therapeutic paradigm for first-line EGFR-mutant advanced NSCLC has evolved from EGFR TKI monotherapy toward intensified combination strategies designed to prolong disease control, delay acquired resistance and ultimately improve OS. In the FLAURA2 trial, the addition of platinum-pemetrexed chemotherapy to osimertinib significantly prolonged mPFS to 25.5 months compared with 16.7 months for osimertinib alone, representing an approximately 10-month improvement, with emerging OS benefit (HR 0.77, 95% CI 0.61–0.96). However, this benefit came at the cost of increased toxicity, including substantially higher rates of grade ≥ 3 adverse events (64–70% vs. 27–34%), cytopenias, fatigue, nausea, and greater treatment complexity due to intravenous chemotherapy administration [86,87,88,89,90,91]. Similarly, the MARIPOSA trial demonstrated that dual EGFR/MET-directed blockade with amivantamab plus lazertinib improved mPFS to 23.7 months compared with 16.6 months for osimertinib monotherapy, with a favorable OS trend (HR 0.75). Nevertheless, this regimen was associated with a distinct toxicity profile, including frequent infusion-related reactions (61–67%), rash, stomatitis, venous thromboembolism, and substantially higher dose modification rates [92,93,94,95,96].

Table 3 summarizes the efficacy, safety, and practical considerations from the major first-line trials in EGFR-mutant NSCLC, highlighting the emerging balance between improved efficacy and increased treatment-related toxicity. However, because FLAURA, FLAURA2, and MARIPOSA differed in study period, eligibility criteria, patient characteristics, control arms, follow-up duration, endpoint assessment, and adverse-event reporting, cross-trial comparisons are descriptive only and should not be considered head-to-head evidence. Treatment effects are best interpreted within the context of each individual randomized trial.

Osimertinib monotherapy remains an effective, convenient, and well-tolerated standard for many patients, particularly older individuals, those with comorbidities, or patients prioritizing quality of life and treatment simplicity. In contrast, treatment intensification may be appropriate for selected high-risk patients when the potential benefits outweigh the increased toxicity, cost, and logistical burden. Clinical and molecular features that may support this approach include younger age, good performance status, high disease burden, CNS metastases, TP53 co-mutations, and other characteristics associated with early therapeutic resistance, where maximizing the depth and duration of response may be particularly desirable. However, validated biomarkers for selecting one first-line strategy over another remain limited [15,16,17,66,87,90,92,94,96,97].

#### 2.2.3. Other Combination Therapies for First-Line and Beyond EGFR-Mutant NSCLC

The success of recent combination strategies in EGFR-mutant NSCLC represents a paradigm shift in the treatment landscape. Before 2024, numerous combination approaches had been investigated; however, most demonstrated limited efficacy or yielded negative results. Several phase III studies evaluating first-generation EGFR TKIs in combination with chemotherapy failed to show meaningful survival benefits in patients with advanced NSCLC harboring classic EGFR-sensitizing somatic mutations [98,99,100,101]. These disappointing outcomes may, in part, be attributable to the distinct biological effects of first-generation EGFR TKIs, such as erlotinib and gefitinib, in EGFR-mutant versus EGFR-wild-type tumors. In classic EGFR-mutant NSCLC, the antitumor activity of first-generation EGFR TKIs is primarily mediated through cytotoxic induction of apoptosis [102,103], whereas in EGFR-wild-type tumors, their effects are predominantly cytostatic, characterized by G1 cell-cycle arrest and inhibition of tumor proliferation [102,103]. Consequently, concurrent administration of chemotherapy and EGFR TKIs raised concerns regarding potential pharmacodynamic antagonism, whereby TKI-induced cytostasis could attenuate the efficacy of cell-cycle-dependent cytotoxic chemotherapy. To address this challenge, several early-phase studies explored pharmacodynamic separation or intercalated scheduling strategies designed to minimize treatment antagonism [104]. In the only randomized phase II trial (NCT00950365), 52 patients with unselected or EGFR-wild-type advanced non-squamous NSCLC were randomized to receive pemetrexed with intercalated erlotinib or pemetrexed alone [105]. The combination arm demonstrated numerically improved efficacy outcomes, including median PFS (5.3 vs. 3.5 months), ORR (29% vs. 10%), 6-month PFS (45% vs. 29%), and 12-month PFS (23% vs. 10%), although none reached statistical significance. Rash and diarrhea occurred more frequently in the combination arm but were generally manageable [105].

Combination strategies targeting angiogenesis and EGFR inhibition are supported by strong preclinical rationale. The EGFR and angiogenic pathways are highly interconnected and share multiple downstream signaling mediators. Notably, increased expression of vascular endothelial growth factor (VEGF) has been observed in patients with acquired resistance to EGFR TKIs, supporting VEGF pathway activation as a potential resistance mechanism [106]. Based on this biologic rationale, combinations of erlotinib with antiangiogenic agents, including ramucirumab or bevacizumab, have been developed as first-line treatment options for patients with EGFR-mutant non-squamous NSCLC [107,108]. However, for the third generation EGFR TKI osimertinib, several early phase II studies failed to demonstrate significant PFS benefit with bevacizumab-containing combinations [109,110,111]. A recent randomized phase II RAMOSE trial demonstrated significantly prolonged median PFS with osimertinib plus ramucirumab compared with osimertinib alone (24.8 vs. 15.6 months; HR 0.55, 95% CI 0.32–0.93; *p* = 0.023) [112]. Differences in ramucirumab dosing schedules across studies have been proposed as a potential explanation for the discordant findings [113].

Currently, second-line treatment for EGFR-mutant NSCLC is highly individualized and depends largely on prior first-line therapy and the underlying resistance mechanisms such as EGFR C797S and MET amplification and/or overexpression, typically identified through tissue and/or plasma NGS [114]. Next-generation EGFR-TKIs and MET inhibitors are being actively investigated for these mechanisms. For patients without actionable resistance alterations after osimertinib progression, treatment options include amivantamab plus chemotherapy [115], platinum-based chemotherapy (e.g., carboplatin plus pemetrexed) if not previously administered, and datopotamab deruxtecan, a trophoblast cell-surface antigen 2 (TROP2)-directed antibody–drug conjugate (ADC), which significantly improved PFS versus docetaxel, particularly in nonsquamous NSCLC [116,117]. In addition, sacituzumab tirumotecan (sac-TMT), another TROP2-directed ADC, improved median PFS compared with chemotherapy (8.3 months vs. 4.3 months; hazard ratio for disease progression or death, 0.49), and OS (hazard ratio for death, 0.60) in Chinese patients, with an 18-month OS of 65.8% and 48.0%, respectively [118]. The results of randomized phase II FLAME study further support early on-treatment plasma ctDNA as a potential tool for adaptive treatment intensification. Among patients with persistent plasma EGFR-mutant ctDNA after 3 weeks of first-line osimertinib, early addition of carboplatin-pemetrexed improved progression-free survival compared with continued osimertinib alone, although with higher rates of grade ≥ 3 treatment-related adverse events and a need for further validation before routine implementation [119]. Together, these developments reinforce that broad, validated tissue- and/or blood-based multigene testing is increasingly used in EGFR-mutant NSCLC as a prerequisite for subtype-specific therapy, resistance profiling, ctDNA-guided treatment adaptation, and equitable global implementation [120].

For patients with oligoprogressive disease, typically defined as progression at a limited number of sites (commonly ≤5 lesions) while the majority of disease remains controlled, local ablative therapies, including radiation or surgery, are frequently employed to prolong the benefit of ongoing systemic EGFR-targeted therapy.

More recently, immunotherapy- and antiangiogenic-based combination approaches have demonstrated encouraging activity in EGFR-mutant NSCLC following EGFR TKI failure. In the phase III IMpower150 trial, the addition of atezolizumab to bevacizumab, carboplatin, and paclitaxel (ABCP regimen) improved mPFS and OS compared with bevacizumab plus chemotherapy in a subgroup of patients with EGFR-mutant NSCLC who had progressed after prior EGFR TKI therapy [121,122]. These findings provided early clinical evidence supporting the potential role of combined vascular endothelial growth factor (VEGF) inhibition and immunotherapy in overcoming resistance to EGFR-targeted therapy. Subsequently, a meta-analysis including 17 single-arm and 15 randomized studies demonstrated that the combination of chemotherapy, immune checkpoint inhibitors (ICIs), and antiangiogenic agents was associated with the most favorable PFS outcomes among available treatment strategies for metastatic EGFR-mutant NSCLC after EGFR TKI failure [123]. More recently, the phase III HARMONi-A trial demonstrated that ivonescimab, a bispecific antibody targeting programmed cell death protein 1 (PD-1) and VEGF, in combination with chemotherapy significantly prolonged PFS compared with placebo plus chemotherapy in Chinese patients with classical EGFR-mutated, locally advanced or metastatic non-squamous NSCLC following EGFR TKI progression (7.06 vs. 4.80 months) [124]. In the parallel global phase III HARMONi study, which enrolled approximately 38% of patients from North America and Europe in addition to Chinese patients, ivonescimab plus chemotherapy significantly improved mPFS compared with chemotherapy alone at a median follow-up of 22.3 months (6.8 vs. 4.4 months; HR 0.52, 95% CI 0.41–0.66; *p* < 0.001) [125]. At a median follow-up of 29.7 months, mOS numerically favored the ivonescimab arm (16.8 vs. 14.0 months), although statistical significance was not reached (HR 0.79, 95% CI 0.62–1.01; *p* = 0.057). The ORR was also numerically higher with ivonescimab plus chemotherapy (44.7% vs. 34.2%). Although immune-related and VEGF-related adverse events occurred more frequently with ivonescimab, these toxicities were predominantly low grade and generally manageable. The extent to which racial and ethnic differences contribute to the observed variability in efficacy across studies remains uncertain, highlighting the need for further investigation in diverse patient populations [125].

## 3. Germline EGFR Mutations

Although tobacco exposure remains the dominant risk factor for lung cancer, 10–20% of all lung cancer cases in the United States and worldwide arises in never-smokers or clusters within families. Some studies suggest that while overall lung cancer rates are declining, the proportion of cases in non-smokers is rising, having increased from about 8% (1990–1995) to nearly 15% (2011–2013) in certain studies. The majority (60–80%) are adenocarcinomas. Key risk factors include passive smoking (secondhand smoke), radon exposure, air pollution, asbestos, and genetic factors. These tumors are often enriched for oncogenic drivers and mainly in individuals who do not meet smoking-based screening criteria. The expanding use of tumor NGS, plasma cell-free DNA profiling, and paired tumor-normal sequencing has changed how hereditary lung cancer is recognized. Inherited susceptibility is now suspected not only from family history, but also from genomic findings generated during routine cancer care [126,127,128,129]. This section summarizes the biology, clinical phenotype, detection workflow, variant spectrum, surveillance uncertainty, and treatment interpretation for germline EGFR-associated lung cancer.

### 3.1. Hereditary EGFR Mutations and Lung Cancer Predisposition

Germline EGFR mutations are rare but are increasingly recognized as contributors to inherited lung cancer susceptibility, particularly among never-smokers and familial clusters [126,127,128,129,130,131,132,133,134,135,136,137,138,139,140]. Germline EGFR T790M is the best-characterized pathogenic variant. Germline EGFR T790M was first described in familial lung cancer nearly two decades ago and remains the canonical hereditary EGFR-associated variant [130,131,132]. The ongoing Investigating Hereditary Risk in Thoracic Cancers (INHERIT) study (NCT05587439) is prospectively evaluating individuals with germline EGFR and other hereditary cancer predisposition variants, as well as families with suspected inherited lung cancer, to better define lung cancer risk, optimize screening strategies, and characterize the natural history of hereditary thoracic malignancies. Early findings from the INHERIT study indicate that most lung tumors acquire a second somatic EGFR driver mutation, supporting a multistep model of tumorigenesis [129]. This distinction is clinically important in the osimertinib era.

Although EGFR T790M is commonly recognized as an acquired resistance mutation following first- or second-generation EGFR TKIs, detection of T790M before treatment should raise suspicion for a germline mutation. In such cases, germline confirmation, genetic counseling, cascade testing of at-risk relatives, and individualized surveillance should be considered, while tumor-directed treatment decisions should continue to be guided by the tumor’s somatic driver alterations, resistance mechanisms, disease stage, and clinical behavior. Pretreatment EGFR T790M is the strongest molecular trigger for germline evaluation. Other molecular, radiographic, and clinical features may increase suspicion but are less specific and should be interpreted collectively, as detailed in Section 3.4 [126].

### 3.2. Pathogenesis and Two-Hit Tumorigenesis

Germline EGFR-associated lung cancer is commonly conceptualized using a two-hit model in which an inherited EGFR variant, most commonly T790M, creates a permissive epithelial background and tumor development is frequently accompanied by acquisition of a second somatic EGFR driver. EGFR T790M substitutes methionine for threonine at the kinase-domain gatekeeper residue. Structurally, this increases ATP affinity and impairs binding of earlier-generation EGFR TKIs, explaining its classic role in acquired resistance [133]. As a germline allele, however, T790M appears to function as a permissive first hit or susceptibility factor rather than a fully penetrant driver. Carriers may develop multiple synchronous or metachronous lung cancers, and tumors commonly acquire a second somatic driver. Functional and mouse-model data suggest that T790M alone has limited oncogenicity but can enhance kinase activity and transformation when paired with activating EGFR alterations such as L858R or exon 19 deletion [134,135,136].

**Figure 3:**
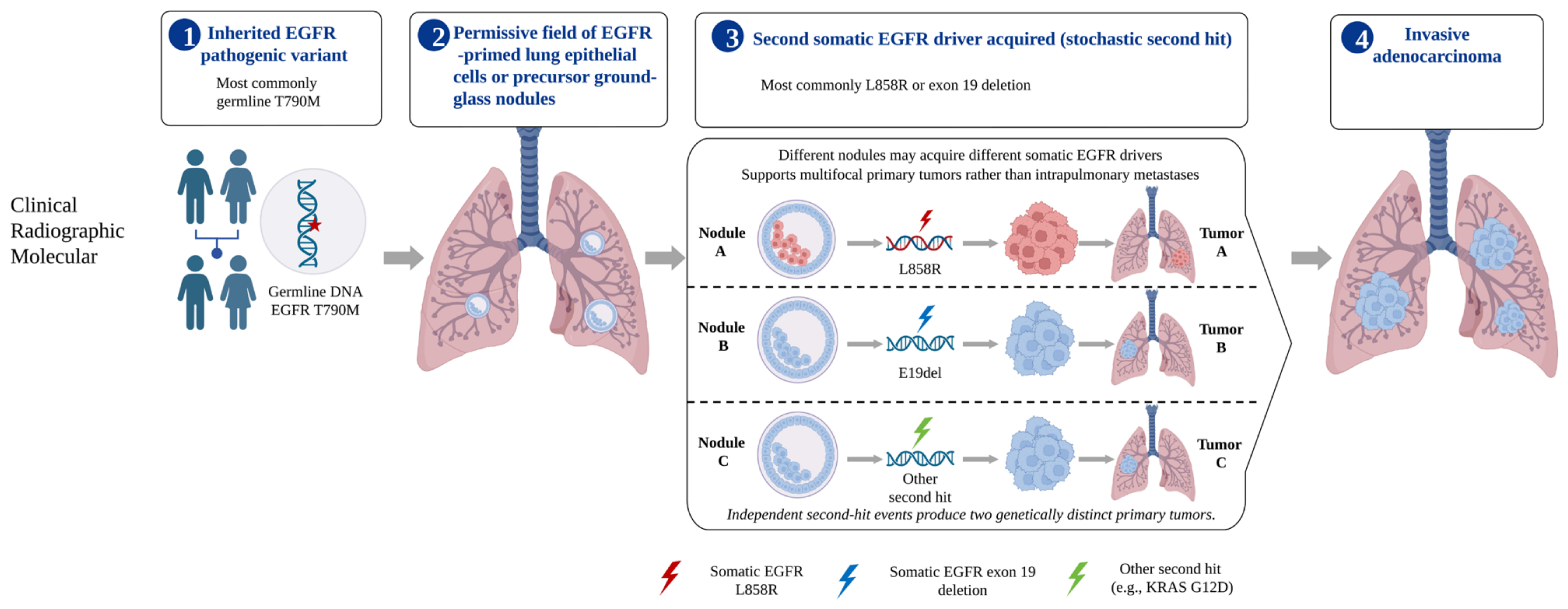
Two-hit tumorigenesis model of germline EGFR-associated lung cancer. An inherited EGFR pathogenic variant, most commonly germline T790M, creates a permissive field of EGFR-primed lung epithelial cells or precursor ground-glass nodules. Acquisition of a second driver event, commonly EGFR L858R or exon 19 deletion in reported cohorts, may contribute to the development of independent primary tumors in separate nodules. The green pathway denotes other oncogenic second-hit events, such as KRAS G12D, that may arise in distinct lesions. This model supports careful interpretation of multifocal disease as potentially separate primary tumors rather than automatic intrapulmonary metastases. However, it is based primarily on familial and clinically selected cohorts and has not been validated in population-based studies.

The two-hit model represents the leading biologic framework for germline EGFR-associated lung cancer, though it is supported primarily by a limited number of familial cohorts, institutional case series, and preclinical models rather than large-scale prospective studies. The inherited variant creates a permissive epithelial field, and progression usually requires acquisition of a second somatic EGFR driver. INHERIT provided the clearest clinical support: 35 of 37 tumors (95%) with available somatic testing harbored a second somatic EGFR driver [129]. This model may also help explain why carriers may develop multiple ground-glass or part-solid nodules. Separate nodules can acquire different somatic EGFR events, supporting synchronous or metachronous primary adenocarcinomas rather than intrapulmonary metastases. Nevertheless, lesion-specific genomic profiling is often unavailable, and distinct somatic alterations do not by themselves definitively establish independent tumor origin. This distinction is clinically important because multifocal early-stage primaries may be managed with local therapy and surveillance, whereas metastatic spread implies different staging and systemic treatment priorities. Clinically, this proposed two-hit framework has several implications. Multifocal nodules in germline EGFR carriers should not automatically be classified as intrapulmonary metastases, because separate lesions may acquire distinct somatic drivers and represent independent primary tumors. Staging should therefore integrate radiographic evolution, histopathology, and lesion-specific molecular findings when available. Germline status may also support individualized surveillance discussions, but it does not currently justify a standardized intensified imaging schedule. Similarly, systemic treatment should be selected according to the tumor’s somatic driver, disease stage, and resistance mechanisms rather than the inherited EGFR variant alone. The proposed two-hit model and its clinical implications for multifocal pulmonary lesions are summarized in Figure 3.

### 3.3. Phenotype and Prevalence

The clinical phenotype combines inherited susceptibility with the familiar molecular pattern of EGFR-driven lung adenocarcinoma. In INHERIT, 91 confirmed or obligate germline EGFR pathogenic-variant carriers from 39 kindreds were identified; 50 (55%) had lung cancer, 15 of 70 evaluable carriers (21%) were diagnosed by age 50, and 34 of 65 (52%) by age 60 [129]. Because participants were identified through affected families and hereditary-risk referral pathways, these proportions describe a family-enriched cohort and should not be interpreted as population-based penetrance estimates. Lung nodules are also common in unaffected carriers. Among cancer-free carriers in INHERIT with CT imaging, 9 of 15 (60%) had pulmonary nodules, and the MD Anderson cohort reported multifocal ground-glass opacities in 72.7% of patients with germline EGFR-associated lung adenocarcinoma [129,137]. These radiographic estimates are likewise derived from small, clinically selected cohorts. Together, the findings support a field-defect model but also highlight incomplete penetrance: some carriers may remain asymptomatic with stable nodules, whereas others develop multifocal invasive disease. A 13-year prospective follow-up of seven confirmed germline EGFR T790M carriers further showed heterogeneous radiographic trajectories, with some carriers developing lung adenocarcinoma while others had bilateral ground-glass opacities or nodules that remained stable or slowly progressive over 6–10 years [138].

True population prevalence remains uncertain because available data are enriched for affected families, institutional testing cohorts, plasma NGS datasets, or paired tumor-normal sequencing studies. Population-reference datasets suggest that germline EGFR T790M occurs in approximately 1 in 3000 to 1 in 7000 individuals, although regional enrichment may exist; O’Brien et al. reported a prevalence exceeding 1 in 3000 in the Duke catchment area [140]. In clinically ascertained cohorts, frequencies have ranged from 0.15% in a large pan-cancer plasma NGS dataset to 2.1% in a single-center Azerbaijani NSCLC cohort with possible family-history enrichment (Table 4) [128,129,137,140,141,142]. This variation likely reflects differences in ascertainment, ancestry, geography, and cohort selection; therefore, these estimates should be considered approximate rather than definitive population prevalence.

### 3.4. Detection Workflow

**Figure 4:**
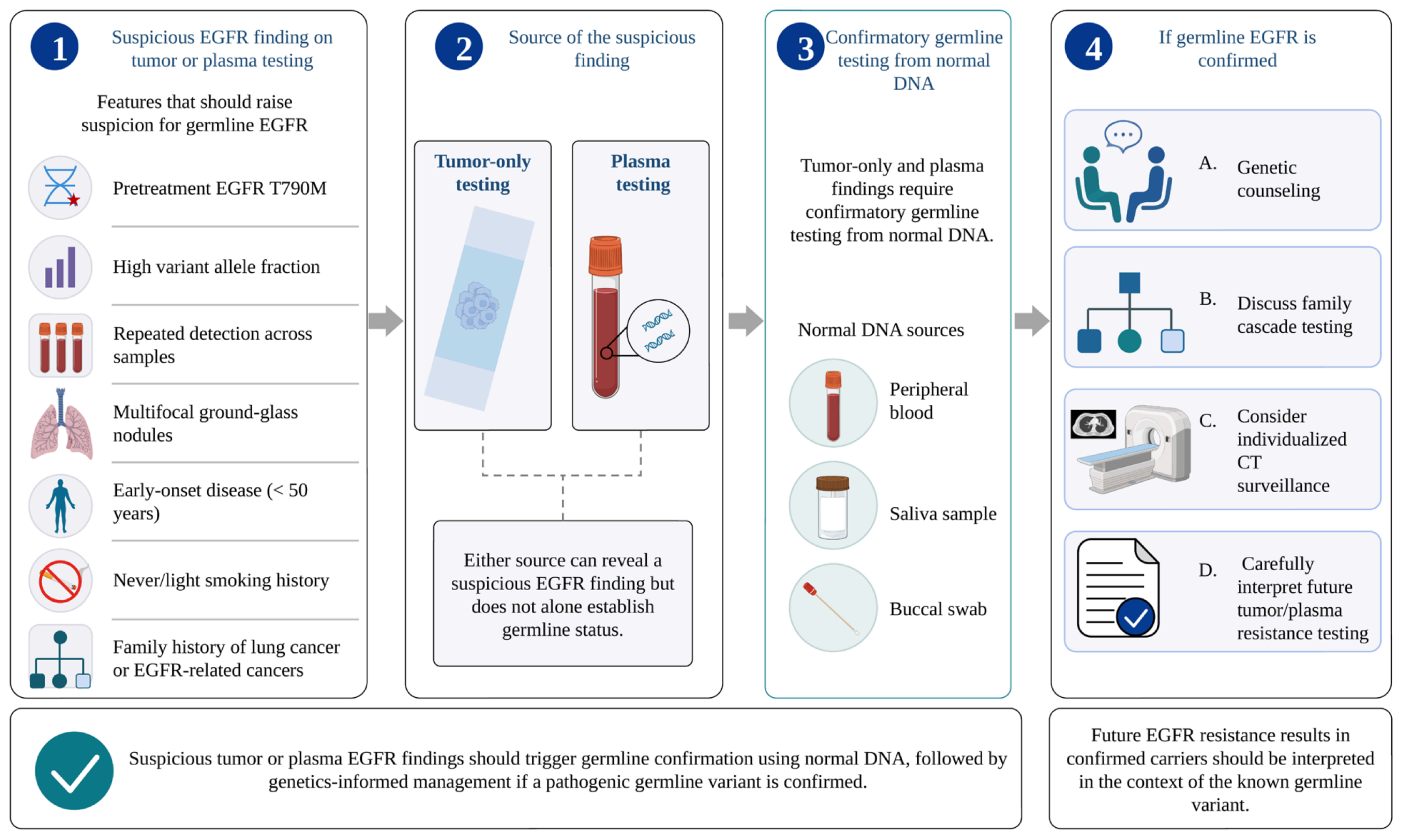
Workflow after a suspicious EGFR finding on tumor or plasma testing. Pretreatment T790M, high variant allele fraction, repeated detection, multifocal ground-glass nodules, early-onset disease, never/light smoking history, or family history should prompt germline consideration. Tumor-only and plasma findings require confirmation from normal DNA before genetics-informed management. Detailed workflow triggers, interpretation caveats, and key references are provided in Supplementary Table S2 [55,56,67,127,128,142,143,144,145,146].

Germline EGFR is usually suspected during tumor or plasma testing performed for treatment selection rather than through dedicated hereditary-risk screening. The strongest trigger for germline evaluation is EGFR T790M detected before exposure to an EGFR TKI. Additional high-suspicion findings include a near-heterozygous VAF, persistent detection despite changes in tumor burden, multifocal bilateral ground-glass nodules, or multiple primary lung adenocarcinomas. Young age at diagnosis, never- or light-smoking history, family history of lung cancer, and other rare EGFR variants are supportive but less specific and should not independently establish germline status. A high allele fraction should prompt germline consideration, but lower or confounded allele fractions do not exclude inheritance, especially in plasma samples affected by tumor fraction, clonal hematopoiesis, or assay-specific factors [127,128,143]. No single feature or VAF threshold is diagnostic; germline testing decisions should integrate treatment history, molecular findings, radiographic phenotype, and personal and family history. A practical stepwise workflow for evaluating suspicious EGFR findings detected through tumor or plasma testing is presented in Figure 4.

Tumor-only and plasma cfDNA testing can raise suspicion but cannot establish germline status. Genetic counseling should be recommended when pretreatment EGFR T790M is detected or when another suspicious EGFR finding occurs together with supportive clinical features, such as multifocal nodules, multiple primary lung cancers, early-onset disease, or a relevant family history. VAF should be interpreted in the context of tumor fraction, disease burden, treatment timing, and assay characteristics; neither a near-50% nor a borderline VAF is diagnostic. Discordant tissue and plasma findings should prompt review of specimen timing, assay sensitivity, and serial results, while plasma-only variants may reflect clonal hematopoiesis or technical artifacts. A concerning EGFR finding should prompt referral to cancer genetics and confirmatory testing from normal DNA, typically using a validated germline assay on blood, saliva, or buccal DNA and interpreted alongside personal and family history. If a hematopoietic source of the variant is suspected, confirmation using an appropriate nonhematopoietic specimen may be necessary. Paired tumor-normal sequencing is more informative than tumor-only testing, but it is not a substitute for a hereditary cancer evaluation unless supported by consent, reporting pathways, confirmatory standards, and genetics infrastructure [142,144]. The INHERITY LC study reported a 10.3% prevalence of pathogenic germline variants across multiple cancer-predisposition genes in a selected NSCLC cohort and showed that cascade testing can identify unaffected relatives carrying pathogenic variants [145]. Consistent with NCCN guidance, EGFR p.T790M detected before EGFR TKI exposure should prompt genetic counseling and consideration of confirmatory germline testing [67]. When a pathogenic germline variant is confirmed, cascade testing should be offered to adult at-risk relatives through genetic counseling, with discussion of uncertain penetrance, surveillance limitations, psychosocial implications, and potential insurance considerations.

### 3.5. Non-T790M EGFR Variants

Non-T790M germline EGFR variants are supported by substantially less evidence than germline T790M and may not confer the same degree of lung cancer susceptibility as germline T790M. For most variants, available evidence is limited to individual families, case reports, small case series, or incomplete functional characterization. Among these variants, V843I has the strongest supporting evidence, with reports of familial lung adenocarcinoma, multiple primary lung cancers, increased EGFR phosphorylation in functional studies, and acquisition of second somatic EGFR driver mutations [147,148,149,150]. R776H and R776G variants also have emerging familial and functional evidence but remain less well characterized [151,152,153,154,155]. In contrast, de novo germline L858R appears to represent a distinct clinical phenotype, characterized by dermatologic manifestations, bilateral pulmonary nodules, and responses of both cutaneous and pulmonary lesions to EGFR inhibition in a small number of reported cases [156]. Other rare germline variants, including A871E, P848L, K757R, G863D, D1014N, and T725M, should be interpreted cautiously because evidence for pathogenicity, including familial segregation, functional validation, and clinical outcome data, remains limited [157,158,159,160,161]. Table 5 summarizes germline EGFR variants with known clinical relevance.

### 3.6. Screening and Surveillance for Lung Cancer

Current U.S. lung cancer screening recommendations are anchored in tobacco exposure, while many germline EGFR carriers are never- or light-smokers and would not qualify for standard low-dose CT (LDCT) screening [163]. This mismatch is one of the most important clinical gaps in hereditary EGFR-associated lung cancer. No guideline currently defines when unaffected germline EGFR carriers should begin LDCT, how often imaging should be performed, or how indolent ground-glass nodules should be managed. Given the high frequency of nodules in reported carriers, CT-based surveillance has been proposed as a reasonable consideration through genetics-informed, multidisciplinary care, but no prospective data currently support a specific screening protocol, and the benefits of surveillance in unaffected carriers have not been demonstrated. Any surveillance decisions should ideally occur within registries or prospective studies designed to generate the evidence needed to inform future guidelines.

Surveillance decisions should balance earlier detection against radiation exposure, false positives, overdiagnosis, anxiety, cost, and downstream procedures. Never-smoker LDCT studies, including risk-adapted screening cohorts from East Asia, show that LDCT can detect early-stage lung adenocarcinoma in selected never-smokers, but these data should not be directly extrapolated to germline EGFR carriers [164,165,166]. Polygenic risk scores and common susceptibility loci may eventually refine never-smoker screening, but current PRS performance is not sufficient to replace targeted germline confirmation, cascade testing, and individualized surveillance for suspected or confirmed germline EGFR carriers [167,168,169,170]. EGFR-directed chemoprevention remains investigational; reported responses of pulmonary nodules to EGFR inhibition in germline L858R carriers are limited to isolated case observations, are variant-specific, and should be regarded as hypothesis-generating only. There is currently no basis for recommending preventive EGFR TKI therapy in unaffected germline T790M carriers outside of a clinical trial [156].

### 3.7. Considerations for Prevention

Treatment of lung cancer in germline EGFR carriers should follow the tumor’s somatic driver alterations, disease stage, and clinical behavior rather than the inherited allele alone. Germline T790M is a background allele and should not be mistaken for acquired resistance, molecular residual disease, or progression, especially when it persists at a relatively stable allele fraction during osimertinib-based therapy. Clinicians should track tumor-specific somatic EGFR drivers, such as L858R or exon 19 deletion, and acquired resistance mechanisms such as EGFR C797S, MET amplification, or histologic transformation when clinically suspected [114]. This distinction will become increasingly important as plasma monitoring and minimal residual disease assays move into earlier-stage NSCLC.

For advanced lung adenocarcinoma arising in germline EGFR carriers, standard EGFR-directed therapy is often appropriate because tumors frequently acquire sensitizing somatic EGFR drivers [129,137]. In the MD Anderson cohort, first- or second-line osimertinib was associated with mPFS of 20.4 months and mOS of 82.0 months, although these results should be interpreted in the context of a small selected cohort [137]. First-line combination strategies developed for metastatic EGFR exon 19 deletion or L858R NSCLC are not germline-specific, but they may be relevant when a germline carrier’s tumor harbors standard sensitizing somatic EGFR alterations. Choice of regimen should consider toxicity, treatment burden, patient goals, and long-term tolerability [11,90,91,92,93].

Multifocal disease requires careful staging and multidisciplinary planning. Bilateral ground-glass or part-solid nodules may represent independent adenocarcinoma-spectrum primaries rather than intrapulmonary metastases. When feasible, profiling separate lesions can help distinguish clonally unrelated primaries from metastatic spread by identifying different somatic EGFR co-mutations [67]. However, profiling multiple lesions may be limited by lesion size, location, procedural risk, and the need to preserve lung function. Molecular findings are supportive but not definitive; therefore, the distinction between independent primaries and metastatic spread should integrate radiographic evolution, histopathology, clinical behavior, and genomic findings. Local therapy should preserve lung function and account for future primary risk. At progression on EGFR-directed therapy, management should follow standard post-osimertinib algorithms, including repeat tissue or plasma molecular profiling to identify acquired resistance mechanisms.

## 4. Broader Germline Predisposition

Inherited lung cancer risk extends beyond EGFR and spans common low-penetrance susceptibility variants, polygenic risk, and rare pathogenic variants in cancer-predisposition genes. Germline EGFR-associated risk is variant-specific and characterized by uncertain penetrance, whereas common susceptibility loci and pathogenic variants in other genes represent distinct inherited-risk pathways. At present, these broader germline findings have not been validated to modify penetrance estimates or surveillance recommendations for germline EGFR variants. GWAS have identified loci relevant to lung adenocarcinoma risk, including regions such as 5p15.33, 3q28, 6p21, and 15q25.1, and East Asian studies have identified inherited background associated with EGFR-mutant lung adenocarcinoma [167,171,172,173]. These findings may eventually support population-level risk stratification, but they should not be conflated with germline EGFR variants, which require a hereditary cancer workflow when clinically suspected.

Broader germline testing studies show that patients with lung cancer can harbor pathogenic or likely pathogenic variants in DNA-repair and cancer-predisposition genes, including ATM, BRCA2, CHEK2, TP53, BRCA1, PALB2, MUTYH, and others [142,145,174,175,176,177,178,179,180]. These findings are important for family counseling and, in selected cases, may influence management, but their therapeutic implications in NSCLC remain exploratory. Available PARP inhibitor data support trial-based investigation rather than routine use outside standard indications or an appropriate study [181,182,183]. Germline TP53 warrants separate attention because radiation-associated second malignancy risk may alter management in Li-Fraumeni syndrome. Table 6 summarizes representative broader germline predisposition studies.

## 5. Summary and Perspectives

While somatic EGFR mutations are established predictive biomarkers for treatment selection, early therapeutic intensification, and resistance-directed treatment sequencing in NSCLC, germline EGFR mutations are receiving increasing attention for their potential role in risk-stratified lung cancer screening and prevention within a shared decision-making framework. Germline T790M remains the best-characterized variant, defined by incomplete penetrance, multifocal pulmonary nodules, familial lung adenocarcinoma, and frequent acquisition of a second somatic EGFR driver mutation. As tumor NGS, plasma ctDNA analysis, and paired tumor-normal sequencing become increasingly integrated into routine clinical practice, the key challenge is to identify germline EGFR variants accurately, confirm their hereditary origin, and incorporate this information into clinical management while recognizing the current limitations of the evidence.

Several priorities should guide future research in the field. Population prevalence should be estimated in geographically and ancestrally diverse cohorts rather than extrapolated from affected families. Prospective surveillance studies are needed to define the optimal age to initiate LDCT, appropriate screening intervals, and radiographic features predictive of disease progression. Mechanistic studies should clarify why some carriers remain clinically stable whereas others develop multifocal invasive lung adenocarcinoma. Management of multifocal pulmonary nodules will require multidisciplinary collaboration among radiologists, thoracic surgeons, radiation oncologists, pathologists, molecular diagnosticians, medical oncologists, pulmonologists, and genetic counselors. International registries should systematically capture unaffected carriers, imaging trajectories, tumor genomic evolution, treatment outcomes, and patient-reported outcomes.

Current evidence also has significant limitations. Most published cohorts are enriched for affected individuals or families referred for molecular testing, potentially inflating penetrance estimates. Many non-T790M germline EGFR variants remain supported only by case reports, small case series, or limited functional evidence. No prospective studies have established the optimal LDCT screening age or surveillance interval for unaffected carriers, and treatment recommendations continue to rely largely on retrospective cohorts. Furthermore, available data are derived predominantly from North American, European, and East Asian populations, limiting generalizability to other ancestries.

From a clinical perspective, pretreatment EGFR T790M should prompt a stepwise workflow: review prior TKI exposure and the molecular context, refer for genetics evaluation and confirmatory normal-DNA testing, discuss cascade testing if germline status is confirmed, and consider individualized surveillance through multidisciplinary care. Because no validated surveillance protocol exists, the uncertainty surrounding screening age and interval should be clearly discussed with carriers and their families. From a scientific perspective, germline EGFR-associated lung cancer provides a human model of field cancerization and oncogene cooperation, in which a permissive inherited background may allow separate nodules to acquire distinct somatic second hits and develop as independent primary tumors. However, this framework does not yet predict which lesions will progress or define the appropriate intensity of surveillance.

## 6. Conclusions

Somatic and germline EGFR alterations have distinct but complementary clinical implications in NSCLC. Somatic EGFR mutations are established predictive biomarkers that guide tumor-directed therapy, treatment intensification, resistance assessment, and molecular monitoring. In contrast, confirmed germline EGFR variants, particularly T790M, primarily inform hereditary-risk assessment, genetic counseling, cascade testing, and individualized surveillance discussions. Systemic treatment in germline carriers should remain guided by the tumor’s somatic driver alterations, disease stage, and acquired resistance mechanisms rather than the inherited variant alone.

## Figures and Tables

**Table 1:** Somatic and germline EGFR mutations.

Types of Mutations	Somatic EGFR Mutation	Germline EGFR Mutation
Origin	Acquired in tumor cells	Inherited in all cells
Prevalence in NSCLC	Common (15–50%)	Rare (1% or less)
Primary Role	Driver of carcinogenesis and drug target	Predisposition to lung cancer
Common Mutations	Classic: Exon 19 del, L858R Uncommon: exon 18 G719X, exon 20 S768I, exon 21 L861Q, etc. EGFR E20ins	T790M (50–70% of reported germline mutation carriers), V843I, R776H, P848L, V769M, etc.
Clinical Implication	TKIs, bispecifics, ADCs	Familial risk, early screening, prevention
Resistance	T790M, C797S; often with MET amplification	Often combined with somatic mutations

**Table 2:** Currently available systemic therapies for NSCLC according to EGFR mutation subtype.

Disease Setting	Unresectable					Resectable	
Type of EGFR Mutation	1L Targeted Monotherapy	1L Targeted Combination	1L Targeted + Chemotherapy	2L Targeted Therapy or ADC	Post-ChemoRT	Resected Stage I–III	Neoadjuvant Stage I–III
Common (classic) EGFR mutations (Ex19del or L858R)	Osimertinib Erlotinib; gefitinib; afatinib; dacomitinib † Icotinib (China, November 2014) † Furmonertinib (China, June 2022) † Limertinib (China, April 2025)	Amivantamab + lazertinib (August 2024) * Afatinib + cetuximab Erlotinib + ramucirumab * Erlotinib + bevacizumab	Osimertinib + platinum/pemetrexed (February 2024)	Amivantamab + carboplatin/pemetrexed after EGFR TKI (September 2024) Datopotamab deruxtecan (June 2025) † Savolitinib + osimertinib (MET amplification; China, June 2025) † Sacituzumab tirumotecan (after EGFR TKI + platinum: China, March 2025; after EGFR TKI: China, October 2025)	Osimertinib	Osimertinib	* Osimertinib or Osimertinib + platinum/pemetrexed (NeoADAURA)
Acquired EGFR T790M resistance mutation	—	—	—	Osimertinib † Furmonertinib (China, March 2021) † Limertinib (China, January 2025)	—	—	—
Atypical/PACC EGFR mutations (G719X, S768I, L861Q; E709X, L747P/S)	Afatinib (FDA, January 2018: G719X, L861Q, S768I) * Osimertinib	* Amivantamab + lazertinib	—	—	—	—	—
EGFR exon 20 insertion (Ex20ins) mutations	—	—	Amivantamab + carboplatin/pemetrexed (March 2024)	Amivantamab (May 2021) Sunvozertinib (China, August 2023; US FDA, July 2025) † Furmonertinib (China, February 2026) † Andamertinib (China, April 2026)	—	—	—

Note: Approval dates refer to the listed indication and treatment setting and are shown as month/year. † China-only approval; * not US FDA-approved for the listed indication/setting. 1L, first line; ADC, antibody-drug conjugate; PACC, P-loop and αC-helix compressing.

**Table 3:** Comparison of first-line monotherapy and combination therapies for classic EGFR-mutant NSCLC.

Trial Information /Endpoint	FLAURA Osimertinib vs. First Generation EGFR TKI (NCT02296125) [11,64,66,97]	FLAURA2 Osimertinib + Platinum-Pemetrexed vs. Osimertinib (NCT04035486) [86,87,88,89,90,97]	MARIPOSA Amivantamab-Lazertinib vs. Osimertinib (NCT04487080) [89,92,94,95,96]
Trial design and baseline characteristics			
Treatment arms	Osimertinib/first generation EGFR TKI (gefitinib or erlotinib)	Osimertinib + platinum-pemetrexed/osimertinib	Amivantamab-lazertinib/osimertinib
No. patients	279/277	279/278	429/429
Median age	64 yr/64 yr; age < 65 yr: 298 (53.6%); age ≥ 65 yr: 258 (46.4%)	61 (26–83)/62 (30–85) yr	64 (25–88)/63 (28–88) yr; age ≥ 75 yr: 51 (12%)/53 (12%); age 65 to <75 yr: 143 (33%)/139 (32%); age < 65 yr: 235 (55%)/237 (55%)
Region/race	Asian: 162/160	Asian: 179 (64%)/176 (63%); non-Hispanic White: 74 (27%)/83 (30%); Black: 2 (1%)/3 (1%); Other: 13 (5%)/10 (4%)	Asian: 250 (58%)/251 (59%); non-Hispanic White: 164 (38%)/165 (38%); Hispanic: 7 (2%)/7 (2%); Black: 4 (1%)/3 (1%)
Smoking	NR	NR	Never-smoker: 130 (30%)/134 (31%)
Metastatic sites	CNS: 61/67; liver: 12/13	CNS: 116 (42%)/110 (40%); liver: 43 (15%)/66 (24%); other: 132 (47%)/142 (51%)	CNS: 178 (41%)/172 (40%); liver: 62 (15%)/72 (17%)
EGFR Mutations	EGFR L858R: 103 (37%)/102 (37%); EGFR exon 19 deletion: 176 (63%)/175 (63%)	EGFR L858R: 106 (38%)/107 (38%); EGFR exon 19 deletion: 169 (61%)/168 (60%); TP53 altered: 46/40; TP53 wild type: 33/34	EGFR L858R: 172 (40%)/172 (40%); EGFR exon 19 deletion: 258 (60%)/257 (60%); TP53 altered: 149 (46.6%)/144 (45.6%); TP53 wild type: 117 (36.6%)/130 (41.1%)
Plasma ctDNA	353 (63%) evaluable overall	308 (73.2%) evaluable	266 (83.1%)/274 (86.7%)
Efficacy			
ORR	80%/76%	83%/76%	86%/85%
PFS/OS	mPFS: 18.9/10.2 mo; mOS: 38.6/31.8 mo	mPFS: 25.5/16.7 mo (gain 9.9 mo); mOS: 47.5/37.6 mo; HR 0.77 (95% CI, 0.61–0.96), *p* = 0.02	mPFS: 23.7/16.6 mo (gain > 12 mo); mOS: NE (42.9–NE)/36.7 (33.4–41.0) mo; HR 0.75 (95% CI, 0.61–0.92), *p* = 0.005
CNS response subset	cFAS: 61 (22%)/67 (24%); cEFR: 22 (36%)/19 (28%)	cFAS: 118 (42%)/104 (37%); cEFR: 40 (33.9%)/38 (36.5%)	CNS metastases at baseline: 178 (41%)/172 (40%)
Safety and practical considerations			
All-grade TRAEs	98%/98%	97%/88%	98%/NR
Grade ≥3 AEs	32%/41%	64–70%/27–34%	75–80%/43–52%
Key toxicities	Rash, diarrhea, dry skin, ILD (~3%)	Rash, diarrhea, dry skin, neutropenia (19%/0–2%), anemia (12%/2%), fatigue, nausea, ILD (~3%)	IRRs (61–67%), rash (60–70%), diarrhea, stomatitis, VTE (37%), ILD (~3%)/rash (40–50%), VTE (10%)
Dose reduction	4%/NR	15%/NR	40%/NR
Discontinuation	13%/14%	12%/7%	10%/3%
Supportive care	Emollient cream; topical steroid creams	Emollient cream; topical steroid creams; cytopenia monitoring; mouthwashes as needed	Emollient cream; topical steroid creams; prophylactic antibiotics; anticoagulation considerations
Schedule	Daily oral tablet; visits every 2–3 months	Daily oral osimertinib plus IV platinum-pemetrexed every 3 weeks/daily oral osimertinib	Daily oral lazertinib plus amivantamab infusion weekly for the first 4 weeks, then every 2 weeks/daily oral osimertinib
Relative financial burden	$/comparator EGFR TKI	$$/osimertinib monotherapy	$$$$/osimertinib

Note: Cross-trial comparisons are descriptive only. Differences in study design, eligibility criteria, baseline characteristics, control arms, follow-up duration, endpoint definitions, and safety reporting preclude direct comparisons among FLAURA, FLAURA2, and MARIPOSA. Efficacy and safety results should be interpreted within each randomized trial. $, Relative cost of treatment, increasing from $ to $$$$.

**Table 4:** Selected peer-reviewed cohorts informing germline EGFR-associated lung cancer.

Study	Cohort/Context	Key Numbers	Clinical/Radiographic Signal	Main Message
Hu et al., 2017 [128]	Large plasma NGS cohort All cancer types N = 31,414	48 likely germline T790M carriers (0.15%); 43 had non-squamous NSCLC	cfDNA pattern/allele fraction raised suspicion	cfDNA can flag possible germline T790M; confirm with normal DNA.
Oxnard et al./INHERIT 2023 [129]	Confirmed/obligate germline EGFR carriers 39 kindreds N = 91	55% developed lung cancer; 95% of tested tumors had a second somatic EGFR driver	52% of evaluable carriers diagnosed by age 60; 60% of unaffected carriers had lung nodules	Characterizes a family-enriched predisposition phenotype; supports two-hit/field-effect model.
Pan et al., 2024 (MD Anderson) [137]	Germline EGFR-associated lung adenocarcinoma N = 22	95.5% had germline T790M; osimertinib mPFS 20.4 mo, OS 82.0 mo	72.7% had multifocal GGOs	Modern radiographic and outcomes dataset.
Melikova et al., 2025 [141]	Single-center Azerbaijani NSCLC cohort N = 507	11 confirmed germline T790M carriers (2.1%)	Positive family history; more often stage I-II than somatic T790M	Suggests international variation; caution for enrichment bias.
O’Brien et al., 2026 (Duke/Southeastern US) [140]	Duke + CATHGEN/All of Us/gnomAD/UK Biobank comparisons	Germline EGFR T790M prevalence >1 in 3000 in Duke catchment area	Enriched relative to external population datasets	Supports regional enrichment/possible founder effect.
Govindan et al., 2026 [142]	Paired tumor-normal sequencing Primary lung cancers N = 11,740	P/LP germline alterations: 4.8% smokers; 5.8% never-smokers	EGFR alterations enriched in never-smoker, somatic EGFR-altered tumors	Places germline EGFR in the broader lung cancer germline landscape.

Note: Cancer frequencies and age-at-diagnosis estimates in familial or clinically selected cohorts reflect ascertainment enrichment and should not be interpreted as population-based penetrance estimates. N, number; NGS, next generation sequencing; GGO, groundglass opacity.

**Table 5:** Clinically relevant germline EGFR and EGFR-family variants reported in lung cancer.

Variant (Evidence Level)	Clinical Clues	Second Hit/Biology	Clinical Takeaway	Refs
T790M (best defined)	Familial LUAD; multifocal GGOs/nodules; pretreatment or high-VAF T790M	Second EGFR driver common, often L858R or exon 19 deletion	Confirm in normal DNA; genetics referral and cascade testing; interpret baseline T790M carefully.	[127,128,129,130,131,132,133,134,135,136,137,138,139,140,141,142,143]
L858R (emerging)	Rare de novo syndrome; skin/hair phenotype; bilateral pulmonary nodules	V834L second hit described	Variant-specific; preventive EGFR inhibition is not established.	[156]
V843I (familial reports)	Familial LUAD; multiple primary tumors	L858R, L861Q, or cis L858R described	Supports two-hit model beyond T790M; TKI sensitivity remains uncertain.	[147,148,149,150]
R776H/R776G (emerging)	Familial or multifocal cases; suspicious VAF	Additional somatic EGFR drivers described	Confirm if suspicious; treatment is case-specific.	[151,152,153,154,155]
Other EGFR variants (limited/uncertain)	A871E, P848L, K757R, G863D, D1014N, T725M	Mostly case-level data	Do not assume T790M-equivalent risk without stronger evidence.	[157,158,159,160,161]
ERBB2/HER2 G660D (comparator)	Familial LUAD in one Japanese family	EGFR-family signaling; not an EGFR variant	Discuss separately from germline EGFR-associated lung cancer.	[162]

Evidence levels: Best defined = multiple independent familial cohorts, functional validation, and prospective registry data; Familial reports = ≥2 independent families with segregation data; Emerging = limited familial or functional data; Limited/uncertain = predominantly case reports without functional validation. Clinical decisions for variants with limited evidence should be made on a case-by-case basis with genetic counseling.

**Table 6:** Broader germline predisposition studies in lung cancer.

Study/Focus	Population	Key Germline Finding	Clinical Relevance
Parry et al., 2017 [175] Early signal beyond EGFR	LUAD cases N = 555	2.5% had pathogenic variants; genes included ATM, TP53, BRCA2, EGFR, PARK2.	Inherited susceptibility in LUAD extends beyond EGFR.
Mukherjee et al., 2022 [174] Paired tumor-normal cohort	Advanced lung cancer N = 5118	4.3% had high- or moderate-penetrance PGVs; biallelic tumor inactivation was frequent.	Germline findings may affect tumor biology, not only family risk.
Peng et al., 2022 [176] Diverse cohort	Chinese lung cancer cohort N = 1794	106/1794 (5.9%) carried P/LP variants; BRCA2 and DDR genes were represented.	Supports ancestry-aware interpretation and more diverse datasets.
Govindan et al., 2026 [142] Large paired tumor-normal cohort	Primary lung cancers N = 11,740	P/LP germline alterations: 4.8% in smokers and 5.8% in never-smokers.	Places germline EGFR within the broader lung cancer germline landscape.
GERMLUNG 2024 [177] Selected/enriched cohort	Selected LUAD N = 201	Approximately one-fifth carried P/LP germline variants.	Clinical selection criteria can enrich for germline predisposition.
INHERITY LC 2026 [145] Testing/cascade workflow	Selected Hispanic NSCLC cohort, N = 145	15/145 (10.3%) had PGVs; cascade testing identified unaffected relatives.	Shows practical value of genetics referral and cascade testing.
Cho et al., 2025 [178] Young-onset disease	Early-onset LUAD N = 348 Comparator: N = 1425 later-onset LUAD	TP53 and BRCA2 GPVs enriched in early-onset LUAD; ALKBH2 identified in larger case–control analysis.	Supports germline evaluation in young-onset LUAD.
GELCC/Liu et al., 2025 [179] High-risk families	Familial lung cancer N = 120	Rare high-penetrance variants identified; pathways included mucin-type O-glycosylation and DDR.	Expands familial lung cancer biology beyond EGFR and canonical DDR genes.

## Data Availability

No new data were created or analyzed in this study. Data sharing is not applicable to this article.

## Supplementary Materials

The following supporting information can be downloaded at: https://www.mdpi.com/article/10.3390/cancers18152417/s1, Table S1: Somatic EGFR mutation prevalence/frequency in selected tested NSCLC or lung adenocarcinoma cohorts; Table S2: Detailed workflow triggers and interpretation caveats for suspected germline EGFR.

## Abbreviations

ABCP	atezolizumab, bevacizumab, carboplatin, and paclitaxel
ADC	antibody–drug conjugate
AE	adverse event
ALK	anaplastic lymphoma kinase
AMP	Association for Molecular Pathology
CAP	College of American Pathologists
cEFR	CNS-evaluable-for-response
cFAS	CNS full-analysis set
cfDNA	cell-free DNA
CI	confidence interval
CNS	central nervous system
CNV	copy-number variation
CT	computed tomography
ctDNA	circulating tumor DNA
DDR	DNA damage repair
DoR	duration of response
EGFR	epidermal growth factor receptor
ERBB2/HER2	human epidermal growth factor receptor 2
Ex20ins	exon 20 insertion
FDA	U.S. Food and Drug Administration
FLC	familial lung cancer
GGO	ground-glass opacity
GWAS	genome-wide association study
HR	hazard ratio
IASLC	International Association for the Study of Lung Cancer
ICI	immune checkpoint inhibitor
ILD	interstitial lung disease
IRR	infusion-related reaction
IV	intravenous
LA/mNSCLC	locally advanced or metastatic non-small-cell lung cancer
LDCT	low-dose computed tomography
LUAD	lung adenocarcinoma
LUSC	lung squamous-cell carcinoma
MET	mesenchymal–epithelial transition factor
mOS	median overall survival
mPFS	median progression-free survival
MRD	molecular residual disease
NCCN	National Comprehensive Cancer Network
NCT	ClinicalTrials.gov identifier
NE	not estimable
NGS	next-generation sequencing
NR	not reported
NSCLC	non-small-cell lung cancer
ORR	objective response rate
OS	overall survival
P/LP	pathogenic or likely pathogenic
PACC	P-loop and αC-helix compressing
PARP	poly(ADP-ribose) polymerase
PD-1	programmed cell death protein 1
PD-L1	programmed death ligand 1
PFS	progression-free survival
PGV	pathogenic germline variant
PRS	polygenic risk score
PV	pathogenic variant
sac-TMT	sacituzumab tirumotecan
TKI	tyrosine kinase inhibitor
TMB	tumor mutational burden
TRAE	treatment-related adverse event
TROP2	trophoblast cell-surface antigen 2
VAF	variant allele fraction
VEGF	vascular endothelial growth factor
VTE	venous thromboembolism
VUS	variant of uncertain significance
