# Supplementary material for: Prevalence and Clinical Implications of Somatic and Germline EGFR Mutations in Patients with Non-Small-Cell Lung Cancer
Source: Cancers (Basel). 2026 Jul 27;18(15):2417. doi: 10.3390/cancers18152417 (PMC13463859; doi:10.3390/cancers18152417)
Supplement: Supplementary file 1 [file cancers-18-02417-s001.zip › cancers-4439110-supplementary.pdf]

Supplementary Materials

Prevalence and clinical implications of somatic and germline EGFR mutations in patients with non-small cell lung cancer

Reference numbers in these supplementary tables correspond to the main manuscript reference list. No supplementary-only references are introduced.

Table S1. Somatic EGFR mutation prevalence/frequency in selected tested NSCLC or lung adenocarcinoma cohorts

These estimates refer to somatic tumor EGFR mutation prevalence or frequency in tested cohorts, not population incidence and not germline EGFR prevalence. Values should be interpreted according to histology, smoking status, disease stage, ancestry, and assay platform.

Region / group	Suggested prevalence to report	Cohort / assay context	Interpretation
Global NSCLC	32.3% pooled	456 studies; 115,815 NSCLC patients; mixed testing methods [5]	Historical global benchmark; not NGS-specific.
EARLY-EGFR global / early-stage	51.0% overall in resected stage I-III NSCLC	EARLY-EGFR study; 601 patients from 14 countries, heavily weighted by Asian enrollment [10]	Useful early-stage benchmark, but not a general global prevalence estimate; regional subsets vary substantially.
Asia overall	~49-51%	Melosky meta-analysis; PIONEER Asian advanced lung adenocarcinoma [6-7]	Best broad estimate for Asian advanced lung adenocarcinoma cohorts.
China	~50-54%	PIONEER mainland China subset; recent Chinese targeted-NGS cohort [8, 38]	Use for Chinese LUAD/advanced adenocarcinoma cohorts; older all-NSCLC estimates may be lower.
South Korea	~50-55%	Recent nationwide real-world nonsquamous NSCLC cohort [9]	Consistent with high East Asian EGFR-mutant nonsquamous NSCLC prevalence.
Taiwan	~46-55%; 63% only in selected single-center NGS cohort	Taiwan guideline and real-world testing cohorts; selected 73-patient NGS cohort [18-21]	Update older 35% estimate; label 63% as selected/small, not population prevalence.
Japan	41.7% in resected non-squamous NSCLC; ~49-51% in LUAD/molecular epidemiology cohorts	National resected registry and Japanese LUAD cohorts [22-23]	Do not use 44.6% from selected CGP cohort as EGFR-only prevalence; that figure reflects all druggable mutations [24].
India / South Asia	~23-34% in broad NSCLC/LUAD cohorts; higher (~38-46%) in selected adenocarcinoma-enriched NGS cohorts	Foundational Indian LUAD cohort; EARLY-EGFR India; large 2024-2025 Indian CGP/NGS cohorts; regional review [25, 39-43]	Use ~23-34% as broad estimate; label higher values by histology enrichment and NGS/CGP selection.
Europe / Western populations	~12-15%; Spain 14.5% by PCR and 19.5% by NGS	Melosky meta-analysis; West-European and Spain ORIGEN cohorts [6, 26-27]	Use as broad Western benchmark; Spain ORIGEN illustrates assay sensitivity effects in early-stage resectable NSCLC.
Canada / French-Canadian	~11.5% in resectable LUAD; 23% in a Canadian reflex-testing early-stage cohort enriched for Asian ancestry	LORD project 50-gene NGS panel; Canadian reflexive testing cohort [51-52]	French-Canadian data are consistent with Western estimates; ancestry composition can shift national estimates.

Region / group	Suggested prevalence to report	Cohort / assay context	Interpretation
Black / African American	~6-8% in meta-analysis/real-world NGS; ~12-22% in selected adenocarcinoma cohorts	Costa meta-analysis; Tempus dataset; LCMC1; ancestry/smoking-adjusted analyses [36, 44-46]	Observed differences are confounded by smoking, sex, histology, and cohort selection; avoid treating race as a biologic proxy.
Hispanic/Latino overall	~22% overall; heterogeneous range in US Hispanic/Latino cohorts	Systematic review/meta-analysis and selected Latino datasets [28, 36-37]	Present as heterogeneous rather than one fixed value.
Latin America overall	26.0% in CLICaP; 39.5% in EARLY-EGFR LATAM early-stage/resected cohort	CLICaP 5,738 NSCLC samples; EARLY-EGFR LATAM resected stage I-III [29-30]	Country, ancestry, assay, and stage strongly affect estimates.
Mexico	~34-37%	CLICaP Mexico 34.3%; Mexican tissue targeted-NGS cohort 36.7% [29, 31]	Useful country-specific example of relatively high Latin American prevalence.
Brazil	~20-25% overall/LUAD; 21% regional RT-PCR cohort; 49.6% in never-smoker LUAD	Brazilian real-world/regional cohorts and never-smoker LUAD cohort [34-35]	Keep never-smoker estimate separate from overall Brazil prevalence.
Middle East / North Africa (MENA)	~15-24% by tissue/PCR series; up to ~30% in selected ctDNA NGS cohorts	Benbrahim meta-analysis; Levant prospective cohort; Moroccan NGS cohort; MENA ctDNA NGS study [47-50]	Intermediate between European and East Asian estimates; heterogeneous across countries and testing platforms.
Sub-Saharan Africa	Extremely limited data; small selected adenocarcinoma cohorts report approximately 20% or higher in tested populations	Small Sub-Saharan African cohorts with limited biomarker testing access [53]	Use to emphasize underrepresentation and data scarcity rather than as a stable regional prevalence estimate; results are likely affected by testing access, histology selection, and referral bias.

Table S2. Detailed workflow triggers and interpretation caveats for suspected germline EGFR

This table expands the clinical workflow shown in Figure 4 of the main manuscript. It provides clinical triggers, interpretation caveats, recommended actions, and reference support for suspected germline EGFR findings.

Clinical trigger	Scenario	Interpretation	Recommended action	Refs
Pretreatment EGFR T790M	T790M detected before any EGFR TKI exposure	Possible germline T790M, especially if VAF high; NCCN recommends genetics referral/germline MGPT consideration when no prior EGFR TKI exposure	Refer to genetics; confirm with germline testing from normal DNA	[65, 121-123]
Plasma cfDNA VAF around 40-60%	T790M allele fraction compatible with heterozygous germline allele	Suggestive but not confirmatory; tumor fraction and copy number can confound interpretation	Review tumor burden and assay report; confirm with germline testing	[122]
Repeated detection across samples	Same EGFR variant detected over time independent of disease status	Increases suspicion for inherited allele	Genetics referral and germline testing	[121-123]
Multifocal GGOs or multiple primaries	Bilateral ground-glass/part-solid nodules; multiple adenocarcinomas	Radiographic field effect; may represent synchronous/metachronous primaries	Consider multidisciplinary review; molecular profiling of separate lesions when feasible to distinguish separate primaries from intrapulmonary metastasis	[123, 125, 131, 134, 136]
Young age, never/light smoker, family history	Early-onset lung adenocarcinoma or familial clustering	Higher pretest probability for hereditary predisposition	Genetics referral; consider broader hereditary cancer panel in addition to EGFR	[65, 120-123, 134-135]
Tumor-only NGS without matched normal	Potential germline-like VAF but no normal comparator	Cannot reliably distinguish germline from somatic; paired tumor-normal is more informative but still not a substitute for dedicated germline evaluation unless appropriate consent/reporting is in place	Do not label as germline; proceed to dedicated germline testing if suspicious	[65, 135, 138, 140, 168]
Confirmed germline EGFR PV	Pathogenic/likely pathogenic EGFR variant in normal DNA	Hereditary lung cancer risk with family implications	Cascade testing; individualized LDCT surveillance discussion; document implications for tumor interpretation	[65, 120-123, 138-139, 157-160]
